# A retrospective study of bovine tuberculosis at the municipal abattoir of Bauchi State, Northeastern Nigeria

**DOI:** 10.14202/vetworld.2018.598-605

**Published:** 2018-05-10

**Authors:** Saleh Mohammed Jajere, Naphtali Nayamanda Atsanda, Asinamai Athliamai Bitrus, Tasiu Mallam Hamisu, Mohammed Dauda Goni

**Affiliations:** 1 Department of Pathology and Microbiology, Faculty of Veterinary Medicine, Universiti Putra Malaysia, 43400 UPM Serdang, Selangor, Malaysia; 2 Department of Veterinary Public Health and Preventive Medicine, Faculty of Veterinary Medicine, University of Maiduguri, P.M.B. 1069, Maiduguri, Borno, Nigeria; 3 Research Unit in Microbial Food Safety and Antimicrobial Resistance, Department of Veterinary Public Health, Faculty of Veterinary Science, Chulalongkorn University, Bangkok 10330, Thailand; 4 Department of Veterinary Microbiology, Faculty of Veterinary Medicine, University of Maiduguri, P.M.B. 1069 Maiduguri, Borno, Nigeria; 5 Department of Microbiology and Parasitology, School of Medical Sciences, Universiti Sains Malaysia, Health Campus, 16150 Kubang Kerian, Kelantan, Malaysia

**Keywords:** Bauchi, bovine tuberculosis, *Mycobacterium bovis*, prevalence, retrospective

## Abstract

**Background and Aim:**

Bovine tuberculosis (bTB) still remains a major zoonotic bacterial disease affecting livestock and humans worldwide. The disease remains a poorly managed tropical disease in most developing countries of the world; where in addition to productivity losses and significance in international trade, it posed a major public health threat to both humans and animals. A retrospective study was designed to investigate the occurrence of bTB lesions at Bauchi municipal abattoir.

**Materials and Methods:**

The study utilized abattoir records spanning a period of 10 years (2004-2013). The records indicated that a total of 1,08,638 heads of cattle comprising *n* = 56,070 males and *n* = 52,570 females were slaughtered at the municipal abattoir during the study period.

**Result:**

Of these heads, *n* = 1230 (1.13%) (95% confidence interval [CI]: 1.07, 1.19) had tuberculous lesions. The annual occurrence during the study period varied significantly (p<0.001) from 0.53% (95% CI: 0.40, 0.67) to 1.87% (95% CI: 1.66, 2.10) in 2010 and 2012, respectively. Females had a significantly higher (p<0.001) prevalence of 2.10% (95% CI: 1.98, 2.23) compared with the males 0.23% (95% CI: 0.19, 0.27). The distribution of suspected gross bTB lesions in different organs showed 11.87% in the lungs, 5.93% in the liver, 1.14% in the heart, and 0.49% accounted for generalized bTB. However, none was observed on the lymph nodes and intestines.

**Conclusion:**

It can be concluded that bTB persists in Bauchi State with annual variations during the study period. This study highlights the importance of meat inspection as an important tool for detecting the presence of bTB lesions.

## Introduction

Animal production in Sub-Saharan Africa is facing a serious challenge due to increased demand for meat, milk, egg, and other animal products as a result of demographic growth, economic development, and urbanization [1]. The existing production system and the utilization of indigenous breeds of cattle cannot satisfy the increasing demands for animal products, thus, the need for intensification of animal husbandry. However, the intensification of animal husbandry and the development of a peri-urban system for livestock production have also led to an increased incidence of many infectious diseases. More than 85% of the cattle population raised in Africa are found in countries where many infectious diseases including bovine tuberculosis (bTB) are not considered as notifiable diseases, and thus, no effort is made toward prevention and control of these diseases [1,2]. In Sub-Saharan Africa and other developing countries, cattle herding serves as a means of livelihoods to many rural communities contributing beef, milk, hides, and skin as well as draft animals. Nigeria has an estimated cattle population of about 13,900,000 million with over 70% of those found in the Northeastern region of the country. However, despite their concentration in this region, cattle production and productivity are largely hampered by diseases particularly bTB [3,4].

bTB is a chronic bacterial zoonotic disease of animals and man caused by *Mycobacterium bovis*, which is characterized by the formation of tubercles mainly in the lungs, intestine, liver, and lymph nodes [5-7]. The disease has a worldwide distribution, and in many countries including Nigeria, bTB remains a major threat to cattle, other domesticated animals and certain populations of wildlife [8,9]. In addition, the World Organization for Animal Health (OIE) classifies bTB as a list-B disease alongside other 116 animal diseases, infections and infestation. OIE listed diseases are considered to be of significant socioeconomic and public health importance with a negative impact on international trade of animals and their products [10,11]. Exposure to aerosols containing *M. bovis* is considered the most frequent route of infection among cattle, but infection by ingestion of contaminated materials has also been reported [12]. In humans, bTB is transmitted through the consumption of unpasteurized milk from infected cow. Thus, the occurrence of bTB in humans is extrapulmonary [13]. bTB is principally a respiratory infection, and a large proportion of the infection is thought to occur through direct transmission of aerosols between animals kept in close proximity [14]. *M. bovis* can also be transmitted through ingestion of the organism from contaminated farm environment. In recent times, *M. bovis* infection in cattle rarely manifests in clinical form; it is now commonly seen in apparently healthy cattle without manifestation of the clinical disease, as such, there is a need for sustained routine surveillance [15]. This is an entirely different situation as to when control programs were first instituted. Bovine tuberculosis still remains a poorly managed disease in animals as well as in human populations in the Sub-Saharan countries [16].

Zoonotic tuberculosis poses a significant public health challenge worldwide, especially in developing countries, Nigeria inclusive where there are deficiencies in prevention and control strategies [12]. The situation is entirely different in developed countries, where significant efforts have been made in the prevention and control of bTB through the implementation of prevention and control strategies such as routine testing of animals, pasteurization of milk, and culling of infected animals [1]. Employing the use of these strategies is vital to the prevention and control of bTB; this is because bTB is considered as a major global public health problem. In addition, studies have shown that the incidence of bTB in humans is underestimated due to the lack of accurate distinction between *Mycobacterium tuberculosis* and *M. bovis*. And since the real incidence of *M. bovis* on human health remains unknown, it is imperative to pursue the advancement of eradication of bTB worldwide with particular interests to be focused mainly in developing countries where the risk of infection with bTB is high [17].

Nigeria shares an extensive border with Chad and Cameroon Republic in the Northeast and Northwestern part of the country, respectively. These borders are porous to the movement of cattle both for market and those in search of grazing land. Previous reports based on abattoir records in Cameroon and Chad Republic revealed the prevalence of bTB as 1.03% and 7.3%, respectively [18,19]. In Nigeria, the prevalence of bTB in slaughtered cattle has been on the increase from 2.5% in 1976 to 5.2% in 2004 [20,21]. In addition, an estimated economic loss per annum due to carcass condemnation as a result of bTB in some selected abattoirs was N13, 871,014 [22], and 14-24 million naira [23]. Despite the reported increase in the prevalence of bTB and losses incurred as a result of carcass condemnation in Nigeria, there are no functional quarantine and disease testing facilities across the borders. This favored interaction of animals from Nigeria, Cameroon, and Chad Republic leading to transmission of disease and consequently surveillance, monitoring, and control of diseases in those animals in Nigeria becomes difficult.

The situation has become such that there are little or no data on the prevalence of bTB for control policy in Nigeria. Detection of bTB in cattle and other susceptible animal species is usually carried out using history of the disease in a herd, clinical and necropsy findings, abattoir meat inspection, and tuberculin skin testing. However, a definitive diagnosis of bTB is made based on bacteriological culture, colonial morphology, biochemical test and molecular detection of *M. bovis* using PCR [13]. In Nigeria, however, such techniques and laboratory procedures have not been fully introduced as diagnostic measures. In addition to this, the best laboratory diagnostic method for definitive diagnosis of bTB is bacteriological culture and isolation of the *Mycobacterium*. This diagnostic technique has not been fully explored in some parts of Nigeria due to its cumbersomeness, long incubation period, the requirement of biosafety level 3 containment, and most often unsafe, inadequate, and ill-equipped laboratories [24]. Thus, postmortem examination of slaughtered cattle during meat inspection in slaughterhouses and use of abattoir records remain the most practical and feasible ways of bTB surveillance and control in Nigeria. This is due to the failure of the Federal government to implement the test and slaughter policy of surveillance, monitoring, and control of bTB in cattle [25]. Intervention measures and early diagnosis to interrupt the transmission of bTB are critical to control, and the effectiveness of testing and culling of infected animals will impact on transmission of the disease. Bauchi State, located within the Northeastern region, is known to harbor and serve as a cattle hub due to its favorable weather conditions and availability of good grazing reserves.

Thus, the current study was designed to investigate the occurrence of bTB gross lesions among slaughtered cattle in the state municipal abattoir utilizing records retrospectively for the period 2004-2013.

## Materials and Methods

### Ethical approval and informed consent

This study was approved by the Research and Ethics Committee of the Faculty of Veterinary Medicine, University of Maiduguri, Nigeria. Approval for data collection was obtained from the Chief Veterinary Officer (CVO) and the Abattoir Manager of Bauchi State, Nigeria.

### Study area

**Figure-1:**
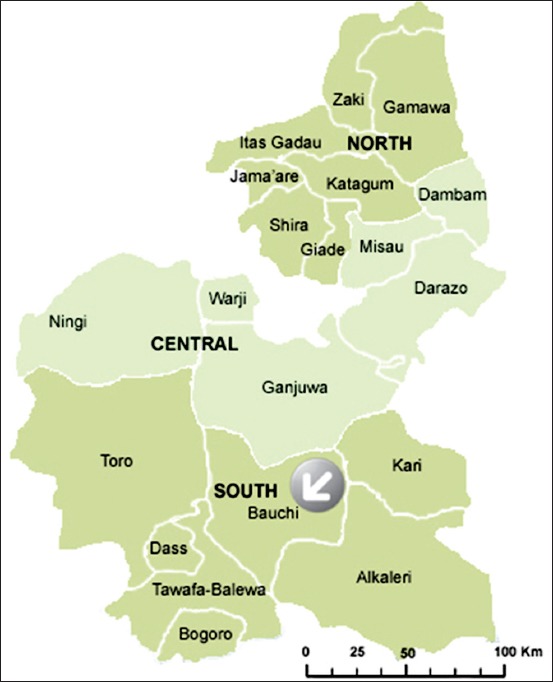
Map of Bauchi State with arrow indicating Bauchi Local Government Area (sourced from Google maps, Accessed on March 26, 2018).

This study was carried out in Bauchi metropolitan, Bauchi State. Bauchi State is one of the 6 states of the Northeastern region of Nigeria. It is located between Latitudes 9°3’ and 12°3’ and Longitude 8°5’ and 11° east. Bauchi State occupies a total land area of 49,119 km^2^ representing about 5.3% of Nigeria’s total landmass (Figure-1). The state shares border with Kano and Jigawa to the North, Taraba and Plateau to the south, Gombe and Yobe to the east, and Kaduna to the west. Bauchi State has an estimated population of 4,653,066 people with 20 local government areas’ councils [26]. The major livestock population found in Bauchi State comprises cattle (1.8 million), sheep (2.8 million), and goats (3.4 million) [27].

### Study design and data retrieval from abattoir records

A retrospective study was carried out to determine the prevalence of bTB in Bauchi municipal abattoir for a 10-year period from 2004 to 2013. Records containing well-documented number of animals slaughtered at the abattoir were duly obtained from the veterinary office situated at the Bauchi abattoir. Daily records of slaughtered cattle during postmortem meat inspection from 2004 to 2013 in the Bauchi State abattoir were collected and examined. The study involved collection of data that described postmortem findings characteristic of tuberculous lesions. Postmortem examination was performed by veterinarians and animal scientists and only cases with lesions typical of TB in the lungs, intestine, liver, heart, and lymph nodes were included in this study.

### Postmortem examination of carcass

A standard daily postmortem examination as described in the OIE manual for meat inspection was the model meat inspection procedure employed and used at the Bauchi municipal abattoir. The procedure is performed by an assigned veterinarian. Lesions characteristics of diseases that can cause devastation to human health such as TB were noted and recorded during the period under study. The postmortem examination of cattle slaughtered at the abattoir was carried out as described by Saidu *et al*. [7]. Briefly, carcass examination was carried out by careful and systematic inspection of the lymph nodes from the head to the abdomen. Other lymph nodes examined includes the superficial and deep popliteal, cervical, and sacral lymph nodes. The procedure also utilizes visual eexamination, palpation and incision of vital visceral organs such as the liver, lungs, kidney, intestinal, and mesenteric lymph nodes.

### Determination of bTB prevalence

In this study, the overall proportion of suspected case of bTB was calculated as the overall total number of bTB cases encountered during the study period divided by the total number of slaughtered cattle examined during the study period. The annual occurrence of suspected cases of bTB was calculated as the overall number of suspected bTB cases recorded in a year divided by the total number of cattle slaughtered and examined in that particular year, while the monthly proportion of the bTB was calculated as the number of suspected bTB cases recorded in a month divided by the total number of slaughtered cattle examined during that particular month.

### Statistical analysis

The data of slaughtered cattle that had suspected bTB granulomatous lesions were extracted from the abattoir records and analyzed in Microsoft office excel^®^ 2007. The data were analyzed using descriptive statistic to show the percentage of animals with suspected bTB lesions. The occurrence of bTB lesions was calculated as described by Thrusfield [28]. The association between the disease and variables such as sex was determined using Chi-square test statistic at 5% level of significance. p*<*0.05 is considered statistically significant.

## Results

**Figure-2:**
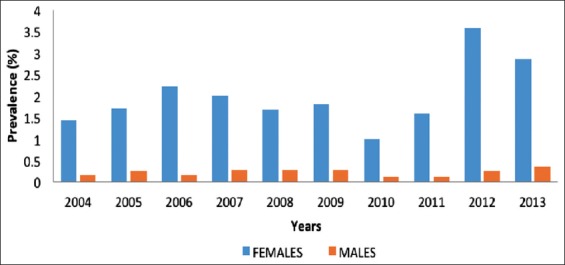
Sex-wise temporal pattern of bovine tuberculosis among slaughtered cattle at Bauchi municipal abattoir (2004-2013).

**Figure-3:**
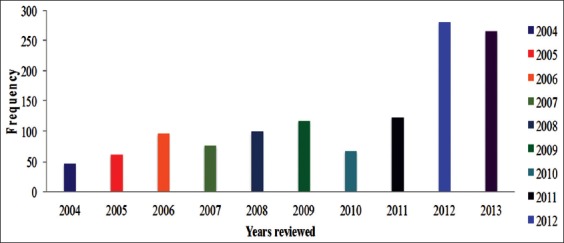
Occurrence of bovine Tuberculosis among slaughtered cattle in Bauchi municipal abattoir, Nigeria (2004-2013).

A total of 108,638 representing 56.07×10^3^ males and 52.57×10^3^ female cattle were slaughtered in Bauchi municipal abattoir from 2004 to 2013. Of these, 1230 had suspected gross bTB lesions representing 1.13% of the occurrence of bTB lesions for the study period (Table-1). The annual distribution of bTB revealed a high prevalence of 1.87% and 1.62% for the years 2012 and 2013, respectively (Table-1 and Figure-3). The lowest prevalence of 0.53% was observed in the year 2010, while prevalence of 0.78%, 0.89%, 1.16%, 1.02%, 0.96%, 1.02%, and 0.84% was observed for the years 2004, 2005, 2006, 2007, 2008, 2009, and 2011, respectively (Table-1). According to sex, females had a significantly higher prevalence (p<0.001) of bTB of 2.01% compared with the males with 0.23% (Table-2). Overall females had higher bTB prevalence compared with the male cattle for all the years (Figure-2). The organ-wise prevalence of bTB revealed the highest prevalence of 11.87% seen in lungs (Table-3), while the prevalence of 5.93%, 0.49%, and 1.14% was observed in the liver, generalized bTB and heart, respectively (Table-3). However, none was observed in the lymph nodes and intestines.

**Table-1:** Annual distribution of gross bTB lesions observed among slaughtered cattle in Bauchi municipal abattoir, Nigeria (2004-2013).

Years	No slaughtered (×10^3^)	Number with suspected bTB lesions	Prevalence* (95% CI)
2004	5.87	46	0.78 (0.58, 1.04)
2005	6.83	61	0.89 (0.69, 1.14)
2006	8.25	96	1.16 (0.95, 1.41)
2007	7.44	76	1.02 (0.82, 1.27)
2008	10.29	99	0.96 (7.97, 11.57)
2009	11.42	117	1.02 (0.85, 1.22)
2010	12.64	67	0.53 (0.42, 0.67)
2011	14.59	123	0.84 (0.7, 1.0)
2012	14.96	280	1.87 (1.66, 2.10)
2013	16.34	265	1.62 (1.44, 1.83)
All years	108.64	1230	1.13 (1.07, 1.19)

* Statistically significant (p<0.001), CI=Confidence interval, bTB=Bovine tuberculosis

**Table-2:** Sex-wise prevalence of bTB gross lesions among slaughtered cattle in Bauchi municipal abattoir, Nigeria (2004-2013).

Sex	No slaughtered (×10^3^)	Number with suspected bTB lesions	Prevalence* (95% CI)
Male	56.07	127	0.23 (0.19, 0.27)
Female	52.57	1103	2.10 (1.98, 2.23)
Total	108.64	1230	1.13 (1.07, 1.19)

* Statistically significant (p<0.001), CI=Confidence interval, bTB=Bovine tuberculosis

**Table-3:** Distribution of suspected gross bTB lesions in different organs of slaughtered cattle in Bauchi municipal abattoir (2004-2013).

Organs	Number of bTB lesions (%)
Lungs	146 (11.87)
Liver	73 (5.93)
Lymph node	0 (0)
Intestines	0 (0)
Heart	14 (1.14)
Generalized bTB	6 (0.49)

bTB=Bovine tuberculosis

## Discussion

bTB is a major animal and human health problem affecting the productivity of the livestock industry worldwide [7,15]. The disease is zoonotic and for many decades poses a serious economic and public health crisis in both developed and underdeveloped countries [17]. The disease is one of the major problems of the cattle industry, due to its negative impact on cattle trade and on the cost and time invested in the genetic improvement of cattle to produce quality traits [15,29,30]. It is against this backdrop, that a retrospective study was conducted to investigate the occurrence of bTB lesions in cattle slaughtered at the Bauchi municipal abattoir, Northeastern Nigeria using postmortem examination of carcass during meat inspection as an instrument of diagnosis. The epidemiological data that we hoped to generate in this study will also serve as a yardstick to assess the effectiveness of meat inspection as a tool in the diagnosis of bTB in Bauchi municipal abattoir. This study reported the occurrence of bTB lesions detected during routine meat inspection at Bauchi municipal abattoir, Bauchi State, spanning a period of 10 years (2004-20013). The overall occurrence of 1.13% obtained in this study is in concordance with other previous reports on the occurrence of bTB lesions in cattle. Cadmus *et al*. [31] reported a prevalence of 1.91% in the Oko Oba abattoir, Lagos State. However, other studies reported a slightly higher prevalence of 2.7% in Maiduguri central abattoir over 10 years’ period [32]. Saidu *et al*. [7] reported an overall prevalence of 0.78% after retrospectively examining a total of 154,562 slaughtered cattle from 2008 to 2015, in Gombe Northeastern Nigeria. The authors also reported high occurrence of bTB lesions in the year 2015 than in 2011, indicating increasing trend in the occurrence of bTB lesions. Interestingly, the occurrence of bTB lesions in this study was observed to have doubled from <1% in 2004 to almost 2% in 2012 and 2013 (Table-1 and Figure-3). This increasing trend in the prevalence of bTB can be as a result of the insurgency that ravaged the region, which seriously affected disease surveillance through routine vaccination of cattle, which is a common practice by the respective government agencies located within these areas. In addition, the insurgency also increases rural-urban migration of people and animals in these areas, which will greatly upset the status of the disease. Even though the overall prevalence of bTB reported in this study is comparatively low, the disease is persistent in Northeastern Nigeria and there is a need for the practice of herd health management program. This will ensure not only the production of apparently healthy cattle for human consumption but also affect the socioeconomic status of the herdsmen as well as families that relied on cattle farming as a means of livelihood. In addition, a number of studies have reported that despite the practice of strict prevention and control measures such as meat inspection, surveillance, vaccination, testing of milk for *M. bovis*, herd testing, and pasteurization of milk, bTB still constitute a major public health problem to humans [15,33-35]. In this regard, there is a need for a concerted effort toward the prevention and control of bTB in Bauchi State.

In this study, females had a significantly higher occurrence of bTB lesions compared with the male cattle (Figure-2). This agrees with the work of Cadmus *et al*. [36] who reported that females had a higher prevalence of bTB compared with the males, probably due to the stress of pregnancy, and are usually kept for a long period of time for breeding purposes. The findings of this study are also in agreement with a cross-sectional study conducted in Uganda using *n*=1470 cattle, in the said study, Inangolet *et al*. [37] reported that the prevalence of bTB following tuberculin skin testing is more in females than in males. This, however, is not in agreement with another cross-sectional study conducted in Tanzania from 1994 to 1997 where a significantly higher prevalence of bTB was reported in male cattle than in females after sampling a total of (*n*=5936) indigenous and exotic breeds of cattle [38]. This shows that the sex of an animal does not have significant effect on the occurrence of bTB lesions in cattle which is in agreement with the report of Clegg *et al*. [39], where the authors reported that the prevalence of bTB was not affected by the sex, or it can be inferred that depending on the region, endemicity of the disease, the number of cattle in a herd, and utilization of the animal. The prevalence of bTB can be significantly affected.

Detection of bTB in Nigeria among slaughtered cattle at abattoir poses a great danger of contracting the disease by abattoir workers and butchers, who wear minimal protective clothing and use bare hands in processing meat of these slaughtered animals. This further confirmed that control measures for bTB are not in place or inadequately applied. This agrees with the works of Corner *et al*. [40], who reported that abattoir monitoring of bTB is the most effective means of detecting residual infection especially in countries that have achieved control of the disease. In addition, Good *et al*. [41] also supported the assertions that having a good national herd program through active abattoir surveillance and tuberculin testing helpful in reducing the occurrence of bTB. This study revealed that 11.87% of the gross bTB lesions were accounted by the lungs. This is lower compared with findings from previous reports that 70-90% of bTB lesions were found in the lungs and lymph nodes of the heads or thoracic cavity [42-45]. This further suggested that the most common route of transmission is through the lungs by aerosol. The annual occurrence of bTB lesions reported in this study varied significantly. The reasons for the fluctuation in the occurrence of bTB lesions for the entire study period could be attributed to inadequacies in capacity and lack of thoroughness of the meat inspectors [13,40]. Several studies have reported that the occurrence of bTB in Nigeria is influenced by changes in the climatic condition; this can also contribute to the fluctuation in the yearly occurrence of bTB in that region [7,35,46]. Pollock and Neill [47] also reported that fluctuations in the occurrence of bTB in Northern Nigeria is dependent on seasonal variation. The movement of cattle through the cattle routes from Northern region to Southern region and back to the Northern region of the country again during the onset of rainy season has been reported to influence the occurrence of bTB lesions in cattle. This is because the animals might have had a latent infection from the South before coming back to the North and vice versa [7,48]. This study further confirmed that bTB persists in Bauchi State, even though annual variation might occur.

It is important to note that other diseases such as Nocardiosis could present similar bacterial nodular lesions as bTB and therefore could be mistaken for bTB during routine meat inspection. Therefore, this signals for proper meat inspection procedures in Nigerian slaughterhouses and abattoirs, which do not have adequate facilities to confirm TB and similar chronic infections [31,49]. Corner [50] reported that when meat inspection procedures are properly carried out, the technique has the ability to identify up to about 95% of animals with visible tuberculous lesions. To reduce the potential zoonotic risk of bTB, there is a need for the maintenance of good abattoir hygiene, the practice of standardized postmortem meat inspection procedures and hygienic handling, and processing of meat in the abattoir and slaughterhouses.

## Conclusion

The result of this study showed that even though the prevalence of bTB is low, the disease persists in Bauchi State and still poses a significant public health risk to humans. There is a need for routine surveillance, regular screening, and vaccination of cattle and other livestock for *M. bovis* in and around Bauchi state. We also recommend increased awareness campaign to herdsmen, farmers, and meat sellers on the zoonotic importance of *M. bovis*.

## Authors’ Contributions

SMJ conceived and designed the study. NNA and AAB conducted and revised the study. TMH analyzed the data and MDG retrieved the data from abattoir records and entered into excel for processing. SMJ, NNA, and AAB drafted and revised the manuscript. All authors read and approved the final manuscript.
